# *Myristica lowiana* Phytochemicals as Inhibitor of Plasmid Conjugation in *Escherichia coli*

**DOI:** 10.1155/2020/1604638

**Published:** 2020-06-19

**Authors:** Awo Afi Kwapong, Sarah Soares, Stephen Ping Teo, Paul Stapleton, Simon Gibbons

**Affiliations:** ^1^Department of Pharmaceutics and Microbiology, School of Pharmacy, University of Ghana, Ghana; ^2^Research Department of Pharmaceutical and Biological Chemistry, UCL School of Pharmacy, University College London, 29-39 Brunswick Square, London WC1N 1AX, UK; ^3^School of Pharmacy, University of East Anglia, Norwich Research Park, Norwich, Norfolk NR4 7TJ, UK

## Abstract

Hexane extract and methanol fraction from the stem bark of *Myristica lowiana* specifically and significantly inhibited the conjugal transfer of the IncW plasmid R7K, a plasmid which harbors ampicillin-, streptomycin-, and spectinomycin-resistant genes. The transfer of this plasmid via the conjugative pilli of *Escherichia coli* was reduced by 76.5 ± 2.0% and 79.0 ± 1.2% by hexane extract and methanol fraction of *M. lowiana*, respectively. The hexane extract exhibited significant anti-conjugant activity at a non-cytotoxic concentration of 100 mg/L as assessed against adult human dermal fibroblast cells. The hexane extract and methanol fraction were screened using phytochemical tests, NMR spectroscopy, IR spectroscopy, and high-resolution electrospray ionization mass spectrometry (HRESIMS) and were found to contain terpenoids, sterols, and fatty acids.

## 1. Introduction

Currently, antibiotic resistance is a major public health concern, especially since to date there is no known universal method for reversing drug resistance in microbes and the world is running out of effective antimicrobials [1, 2]. There is considerable evidence of resistance against almost all existing classes of antimicrobial agents, namely, the *β*-lactams, aminoglycosides, chloramphenicol, glycopeptides, quinolones, oxalidinones, sulfonamides, tetracyclines, macrolides, ansamycins, streptogramins, and lipopeptides [3]. Additionally, it has been predicted that if no antimicrobials or means of reversing antimicrobial resistance are found soon, the death toll for antimicrobial-resistant associated infections may rise to 300 million by 2050 [4]. This illustrates the gravity of antibiotic resistance and the urgent need to address the problem.

All existing antimicrobial agents interfere with essential bacterial growth and metabolic processes or the integrity of the cell. Unfortunately, targeting such vital functions introduces selection pressure for resistant strains and promotes evolution of resistance. The evidence for this is apparent with the rise of antibiotic resistance in hospitals and communities, most notably with organisms such as methicillin-resistant *Staphylococcus aureus*, vancomycin-resistant *Enterococcus*, carbapenem-resistant Enterobacteriaceae (including *Escherichia coli*), multidrug-resistant *Acinetobacter*, drug-resistant *Campylobacter*, multidrug-resistant *Pseudomonas aeruginosa*, drug-resistant *Streptococcus pneumoniae*, drug-resistant *Neisseria gonorrhoeae*, and multidrug-resistant and extensive drug-resistant *Mycobacterium tuberculosis* [5].

A complementary approach of tackling antibiotic or multidrug resistance is to target bacterial adaptation and persistence mechanisms such as horizontal DNA transfer, mutation, antibiotic tolerance, and the production of virulence factors. Such mechanisms are known to be nonessential to the growth of bacteria and as a consequence of this, they are not expected to extensively promote evolution of bacterial resistance against such complementary mediators [6]. The approach of inhibiting bacterial adaptation and persistence mechanisms and the progress made so far have been reported by Fernandez-Lopez et. al. [7], Smith and Romesberg [6], Getino et al. [8], and Getino et al. [9].

In this study, extract and fraction from *Myristica lowiana,* a dioecious flowering tree of the family Myristicaceae, were investigated for their capacities to inhibit plasmid conjugation in *E. coli* a process that utilizes bacterial type-IV secretion systems to transfer genetic material, including antimicrobial resistance genes, between donor and recipient cells [10]. In addition, the extract and fraction were investigated for their antibacterial activities, cytotoxicity, and their phytochemical constituents.

*M. lowiana* is commonly found in Southeast Asian riverine forests. It usually grows up to 7–25 meters in height and has a dark chocolate or blackish coloured stem bark, buttress roots, lanceolate olivaceous or brown leaves, cream rusty coloured flowers, and ellipsoidal-shaped fruits and seeds [11]. *M. lowiana* has a characteristic watery red sap, which oozes out upon cutting its bark and wood. There is no known medicinal use of this plant in the literature, and so far, it is used mainly for construction purposes. There are also no phytochemical reports on this species, but Myristicaceae plants are well known for essential oils, coumarins, flavonoids, lignans, acylphenols, alkaloids, polyketides, and terpenoids [12–15]. Herein, we report on the hexane extract and methanol fraction of *M. lowiana* and their phytochemistry and biological activities.

## 2. Materials and Methods

### 2.1. Plant Material

The stem bark of *M. lowiana* was collected from St. Matang, Malaysia, in May 2014. Stephen Teo, a member of the Forest Department Sarawak identified the species. A voucher specimen (ST001/14) of this tree has been deposited within the herbarium at the UCL School of Pharmacy.

### 2.2. Extraction and Fractionation

Powdered *M. lowiana* stem bark (592 g) was subjected to ultrasound-assisted extraction and extracted successively with hexane, chloroform, and methanol. The powdered plant material was placed in a 5 L glass beaker and covered with the extracting solvent (2.5 L), hexane. This was then placed in an ultrasonic bath for 4 h. The resulting extract was filtered and dried under vacuum. The marc was then dried to remove all residual solvents. The procedure was repeated with the dried marc and chloroform and finally with methanol to obtain extracts of increasing polarity. This yielded a 0.7% hexane extract, a 1.1% chloroform extract, and a 2.2% methanol extract. The extracts were further fractionated using the solid phase extraction. Nonpolar extracts were subjected to normal-phase solid phase extraction (Phenomenex® Strata Silica S1) using a step gradient system, 10% chloroform increments in hexane. Polar extracts were subjected to reverse-phase solid phase extraction (Phenomenex® Strata Silica C18) using a step gradient system, 10% methanol increments in water.

### 2.3. Phytochemical Screening

The extract and fraction of *M. lowiana* were subjected to qualitative phytochemical screening as per the standard methods. For the test of sterols, the test samples were treated with chloroform and concentrated sulphuric acid. A red colouration at the upper layer and a yellow with green fluorescence at the sulphuric acid layer indicated the presence of sterols. For the test of terpenoids, the test samples were treated with acetic anhydride and concentrated sulphuric acid; formation of blue green rings indicated the presence of terpenoids. The biuret test was used to test for the presence of amide bonds; aqueous solutions of the test samples were treated with 2 drops of 2% copper (II) sulphate solution and 1 mL of 5% sodium hydroxide solution. A violet or bluish violet or pink colouration indicated the presence of amide bonds. For flavonoids, the test samples were treated with a few drops of 1% lead acetate; a yellowish precipitate indicated the presence of flavonoids. For saponins, the froth test was used. The test samples were diluted with distilled water and shaken; formation of foam indicated the presence of saponins. For reducing sugars, Benedict's test was used. For tannins, the gelatin test was used where the test samples were treated with 1% gelatin solution containing sodium chloride. Formation of a white precipitate indicated the presence of tannins.

### 2.4. General Experimental Procedures

The 1D- and 2D-NMR spectra were recorded on a Bruker Avance 500 MHz spectrometer. IR spectra were recorded as a thin-film on a PerkinElmer Spectrum 100 FT-IR Spectrometer. Mass spectra were recorded on a Micromass Q-ToF Premier Tandem Mass Spectrometer.

### 2.5. Biological Activities

#### 2.5.1. Materials and Reagents

Norfloxacin, tetracycline, ciprofloxacin, amoxicillin, kanamycin sulphate, nalidixic acid, streptomycin, novobiocin, gentamicin, amphotericin B, linoleic acid, dimethylsulfoxide, 3-[4,5-dimethylthiazol-2-yl]-2,5-diphenyltetrazolium bromide (MTT), trichloroacetic acid (TCA), acetic acid, Tris buffer, sulforhodamine B (SRB), RPMI-1640 medium (AutoMod™), L-glutamine, glucose, bicarbonate of soda, and MOPS [3-(*N*-morpholino)propanesulfonic acid] buffer were purchased from Sigma-Aldrich (Poole, UK). Muller Hinton broth (MHB), Luria Bertani broth (LB), MacConkey agar, nutrient agar, Sabouraud dextrose agar, sodium chloride, sodium hydroxide, phosphate buffered saline (PBS) and human dermal fibroblasts, and adult (HDFa) cells were obtained from Fischer Scientific UK Ltd. Dulbecco's Modified Eagle's Medium (DMEM), Fetal Bovine Serum (FBS), and nonessential amino acids were obtained from Gibco. *Staphylococcus aureus* XU212, which possesses the TetK tetracycline efflux pump and *mecA* gene, was provided by Udo [16]. *S. aureus* 1199B, which possesses the NorA efflux pump, was provided by Kaatz [17]. Dr. Paul Stapleton, UCL School of Pharmacy, provided all other bacterial and fungal strains used in this study.

#### 2.5.2. Antibacterial Activity

The antibacterial activity was determined using the broth microdilution method as described previously [18]. Bacterial strains were cultured on nutrient agar (Fluka Analytical) and incubated at 37°C for 18 hours. A bacterial suspension equivalent to 5 × 10^5^ cfu/mL was added to MHB and the test sample, which had been serially diluted across the 96 well microtitre plate. MICs were determined after 18 hours of incubation at 37°C. With the addition of 20 *μ*L of a 1 mg/mL methanolic solution of MTT, a colour change of the dye to purple indicated the presence of growth. The MIC was defined as the lowest concentration at which no bacterial growth was observed. MIC values were determined in duplicate per plate and repeated in at least two independent experiments.

#### 2.5.3. Antifungal Activity

The antifungal activity was determined using the broth dilution method as described previously [19]. Fungal strains were cultured on Sabouraud dextrose agar plates. A fungal suspension equivalent to 1–5 × 10^5^ cfu/mL was added to supplemented RPMI-1640 and test sample, which had been serially diluted across the 96 well microtitre plate. Control compounds included the known antifungal agents itraconazole and amphotericin B. The MIC was visually determined under light after incubation at 37°C for 24 hours and 48 hours for *C. albicans* and *C. tropicalis*, respectively. MIC values were determined in duplicate per plate and repeated in at least two independent experiments.

#### 2.5.4. Bacterial Strains and Plasmids

Plasmid-containing donor *E. coli* strains WP2, K12 J53-2, and K12 JD173 and recipient *E. coli* strains ER1793 (streptomycin-resistant) and JM109 (nalidixic-resistant) were used in the bacterial conjugation assays. Conjugative plasmids used were pKM101 (IncN; ampicillin-resistant), TP114 (IncI_2_; kanamycin-resistant), and R7K (IncW; ampicillin-, streptomycin-, and spectinomycin-resistant), which were purchased from Deutsche Sammlung von Mikroorganismen und Zellkulturen (DSMZ), and conjugative plasmid pUB307 (IncP; ampicillin-, kanamycin-, and tetracycline-resistant) was provided by Prof. Keith Derbyshire, Wadsworth Center, New York Department of Health.

#### 2.5.5. Bacterial Plasmid Conjugation Inhibition Assay (Liquid Conjugation)

The donor cells with plasmids pKM101, TP114, and pUB307 were mated with the recipient ER1793, while plasmid R7K donor cells were mated with the recipient JM109 in the assay. Conjugation was performed as described previously [20], with minor modifications. Twenty microliters of predetermined inocula (cfu/mL; Supplementary Table 1) of donor and recipient was mixed with 160 *μ*L Luria Bertani (LB) broth and extract or control drug. This was incubated at 37°C for 18 hours after which the number of transconjugants and donor cells were determined using appropriate antibiotic-containing MacConkey agar plates. Linoleic acid, a known inhibitor of IncW plasmid conjugation, was used as a control in this experiment. The extract and fraction were evaluated for anti-conjugant activity at subinhibitory concentration (one-quarter of their MIC against *E. coli* NCTC 10418). Antibiotics and the concentrations used in MacConkey agar for positive identification of donors, recipients, and transconjugants (*μ*g/mL) were amoxicillin (30), streptomycin sulphate (20), nalidixic acid (30), and kanamycin sulphate (20 and 30). Conjugation frequency was calculated as the ratio of total number of transconjugants (cfu/mL) to the total number of donor (cfu/mL) and expressed as a percentage relative to the negative control. This experiment was performed as duplicate in three independent experiments and anticonjugation activity has been reported as mean ± standard deviation.

#### 2.5.6. Cytotoxicity Activity

The sulforhodamine B (SRB) assay as described previously [21] was adapted for the cytotoxicity screening. Adult human dermal fibroblast cells (HDFa; C-013-5C) were grown at 37°C in a humidified atmosphere of 5% in a culture flask (75 cm^2^) which contained Dulbecco's Modified Eagle's Medium, modified with 10% FBS, 1% nonessential amino acids, and 0.1% gentamicin and amphotericin B. The grown cells were seeded in a 96 well microtitre plate, and a determined quantity of test samples was added. The samples were then incubated for 72 h at 37°C in 5% CO_2_. Afterwards, 50 *μ*L of cold 40% ^W^/_V_ trichloroacetic acid (TCA) solution was added and incubated for an hour at 4°C and washed four times with distilled water. Cultured cells were then stained with 0.4% ^W^/_V_ SRB solution and left for an hour at room temperature. Afterwards, the plate was rinsed four times with 1% acetic acid and left for 24 h to dry. Prior to optical density (OD) determination at 510 nm using a microtitre plate reader (Tecan Infinite® M200), 100 *μ*L of 10 mM Tris buffer solution was dispensed into the wells and agitated in an orbital shaker for 5 min, to allow solubilisation of SRB-protein complex. The percentage of viable cell was calculated using the following formula:

(1) $$\begin{array}{c} \text{percentage of viable cell }=\;\frac{\text{OD of test sample}-\text{OD of blank}}{\text{OD of control}-\text{OD of blank }}\;\times \;100. \end{array}$$

This experiment was performed as triplicate in three independent experiments, and cytotoxicity has been reported as mean ± standard deviation.

#### 2.5.7. Statistical Analyses

The statistical analyses were carried out using Excel Data Analysis and GraphPad Prism. Student's *t*-test was used to evaluate the difference between the control conjugal transfer frequency and the test compounds. Results with *p* < 0.05 were considered statistically significant.

## 3. Results and Discussion

In this study, extracts and fractions from *Myristica lowiana* were investigated for their capacities to inhibit plasmid conjugation in *E. coli.* Extract and fraction that showed potent anti-conjugant activity, that is, at least 70% reduction in the transfer frequency of the any of the test plasmids (pKM101, TP114, pUB304 and R7K) was further investigated for their antibacterial activities, cytotoxicity, and phytochemical constituents. The hexane fractions, chloroform extract and its fractions, and methanol extract and its other fractions did not exhibit potent anti-conjugant activity; hence, these products were not further investigated. Some of the aforementioned extracts and fractions also showed no conjugal inhibition, while others showed increase in the conjugation frequency of the test plasmids. The hexane extract and methanol fraction eluted with methanol and water (9 : 1, v/v) were the only products that exhibited potent anti-conjugant activity.

The hexane extract (4.14 g) gave rise to an olive green liquid. Phytochemical screening of this extract revealed the presence of sterols, terpenoids, and amide bonds. However, the extract tested negative for tannins, saponins, reducing sugars, and flavonoids. The NMR spectra (Supplementary Figures 1 and 2), HRESIMS (Supplementary Figure 3), and IR spectrum (Supplementary Figure 4) of the hexane extract indicated the presence of aromatic and aliphatic groups attributable to aromatic compounds, terpenoids, and fatty acids.

The methanol fraction (24.1 mg) was eluted with methanol and water (9 : 1, v/v) to yield a brown solid. Phytochemical screening of this fraction revealed the presence of tannins and amide bonds. This fraction tested negative for saponins, sterols, reducing sugars, flavonoids, and terpenoids. The NMR spectra (Supplementary Figures 5 and 6), HRESIMS (Supplementary Figure 7), and IR spectrum (Supplementary Figure 8) of the methanol fraction indicated the presence of aromatic and aliphatic groups attributable to aromatic compounds and fatty acids.

The antibacterial activities (Table 1) revealed that both hexane extract and methanol fraction had moderate antibacterial activity (32 to 64 mg/L) against Gram-positive bacteria but were not active against Gram-negative organisms. We suggest that the difference in activities against Gram-positive and Gram-negative bacteria may be due to the differences in the cell wall and the additional outer membrane in Gram-negative organisms restricting cell entry or accumulation of components within the extract and fraction [22].

The presence of efflux pumps in *Staphylococcus aureus* strains SA-1199B (NorA pump) and XU212 (TetK pump) did not have any effect on the antibacterial activities of the hexane extract and methanol fraction. This indicated that antimicrobial compounds within the extract and fraction were not susceptible to these prevalent efflux pumps or that the target(s) lies externally to the cell.

**Figure 1:**
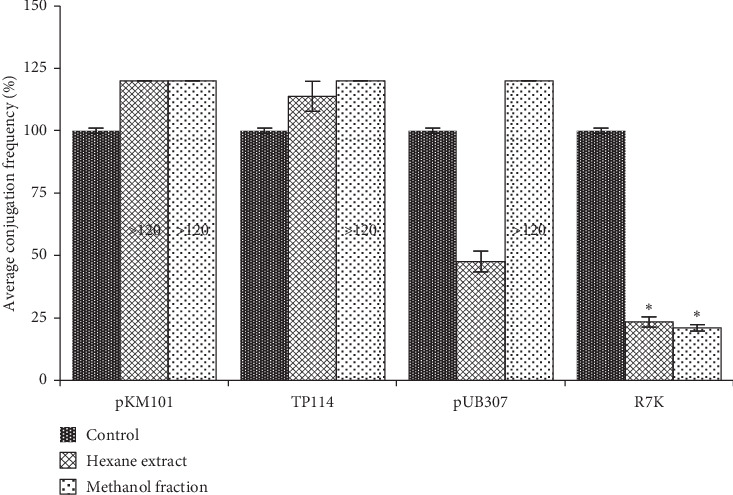
The effect of hexane extract and methanol fraction of *M. lowiana* on the conjugal transfer of plasmids pKM101 (IncN), TP114 (IncI_2_), pUB307 (IncP), and R7K (IncW), expressed as a percentage relative to a control (without extract). The extracts were tested at subinhibitory concentrations, 100 mg/L. ^*∗*^*p* < 0.05 (means significantly different from control).

The extract and fraction were further screened at a sub-inhibitory concentration (100 mg/L) for anti-conjugal activity in *Escherichia coli*. This test concentration was used to ensure that any observed anti-conjugal activity may not be due to growth inhibition. Both hexane extract and methanol fraction showed significant anti-conjugal activity against the transfer of the IncW plasmid R7K, reducing the transfer frequency by 76.5 ± 2.0% and 79 ± 1.2%, respectively (Figure 1). The anti-conjugation activities of the extract and fraction were specific to the IncW plasmid R7K; they did not significantly inhibit the conjugal transfer of IncN plasmid pKM101, IncI_2_ plasmid TP114, and IncP pUB307, rather the extract and fraction increased the conjugation frequency of plasmids pKM101 and TP114 by a further 20% or more. We therefore suspect that the extract and fraction might be targeting different sites on the conjugation machineries, which may be plasmid incompatibility group specific; hence, the difference in activities. This is plausible, as published literature has shown component variations in the conjugation machineries and the mobilizable (MOB) and mating pair formation (Mpf) systems between different plasmid incompatibility groups [8, 9, 23, 24]. The cytotoxicity studies of hexane extract and methanol fraction against adult human dermal fibroblast cells (Supplementary Table 2) revealed that their cytotoxic-IC_50s_ were 154.0 mg/L and 92.6 mg/L, respectively. A comparison of cytotoxicity of the extract and fraction with effective anti-conjugative activities revealed that the cytotoxic-IC_50_ level of the hexane extract was above the concentration (100 mg/L) needed to reduce conjugal transfer of the IncW plasmid R7K by 76.5 ± 2.0%. This suggested that the hexane extract showed anti-conjugal activity at a non-toxic concentration. For the methanol fraction, the cytotoxic-IC_50_ level was slightly below the effective anti-conjugation concentration (100 mg/L), which indicates that cytotoxicity may be an issue for one or more components in the fraction, but whether there is an association with inhibition of plasmid transfer is yet to be determined. No antibacterial activity was observed at a concentration of 512 mg/L for the methanol fraction against *Escherichia coli*. We propose that the activities of the extract and fraction might be through disruption of the conjugative machineries in a highly specific manner, and the slight variation in their anti-conjugal activities against IncW plasmid R7K may be due to the variations in their chemical structure.

**Figure 2:**
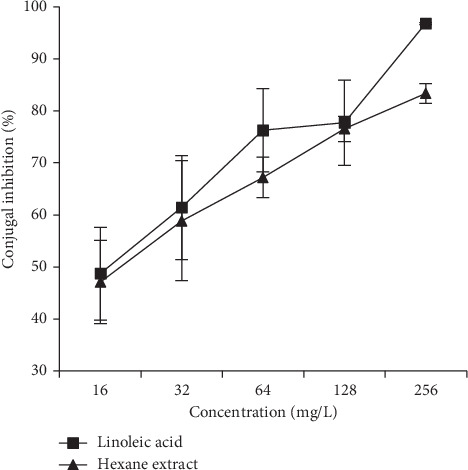
The effect of varying concentrations of hexane extract and the known plasmid transfer inhibitor linoleic acid on the conjugal transfer of the IncW plasmid R7K. The values represent the mean conjugal inhibition frequency ± standard deviation (%).

The hexane extract, which exhibited good anti-conjugant activity at a non-cytotoxic concentration was further evaluated against plasmid R7K (IncW) at sub-inhibitory concentrations, 16 to 256 mg/L. The results (Figure 2) showed that the activity of the hexane extract was concentration dependent and its anti-conjugant activity was comparable to the known IncW plasmid R7K conjugal inhibitor [7], linoleic acid, although the activity of the linoleic acid was slightly more potent. The anti-conjugant IC_50_ of the hexane extract and linoleic acid was found to be 77.8 and 60.5 mg/L, respectively, against the conjugal transfer of IncW plasmid R7K. Statistically, the activities of both the *M. lowiana* hexane extract and linoleic acid were found to be similar since the corresponding *p* value (0.68) indicated that their IC_50_'s were not significantly different.

## 4. Conclusion

These findings show that *M. lowiana* possesses potent anti-conjugant and antimicrobial phytochemicals that are worthy of further explorative investigation. The development a potent anti-conjugant molecule and/or new antimicrobial molecule with a new mechanism of action has the potential in reducing transfer and spread of resistance and virulence within important Gram-negative organisms such as *E. coli*.

## Figures and Tables

**Table 1:** Minimum inhibitory concentrations of hexane extract and methanol fraction from *M. lowiana*.

Strain	MIC (mg/L)						
	Hexane extract	Methanol fraction	Ciprofloxacin	Tetracycline	Norfloxacin	Itraconazole	Amphotericin B
*S. aureus* ATCC 25923	32	32	0.5				
*S. aureus* 13373	64	64	0.5				
MRSA 346724	32	32	>32	<1			
MRSA 274819	64	64	>32	<1			
MSSA 346702	32	64	>32	<1			
*S. aureus* 1199B^a^	32	32	16		32		
*S. aureus* XU212^b^	64	64		128			
*E. faecalis* 12697	32	32	1	32			
*E. coli* NCTC 10418	>512	>512	<0.0625		1		
*P. aeruginosa* 599	>512	>512	0.25				
*P. aeruginosa* 10662	>512	>512	0.25				
*K. pneumoniae* 342	>512	>512	>32				
*K. pneumoniae* 17	>512	>512	>32				
*C. albicans* ATCC 66027	512	n.t^c^				0.125	0.25
*C. tropicalis* ATCC 750	32	n.t^c^				0.125	0.25

^a^ *S. aureus* 1199B overexpresses the *NorA* efflux pump and possesses a gyrase mutation; both contribute to high level resistance to fluoroquinolones. ^b^*S. aureus* XU212 overexpresses a *TetK* efflux pump that confers resistance to tetracycline and carries the *mecA* gene that gives rise to beta-lactam resistance. ^c^n.t, fraction not tested.

## Data Availability

All data generated or analysed during this study are included in this published article and its supplementary information file.

## Supplementary Materials

Supplementary Materials Table 1: the conjugation pair, their average population size used, and their average conjugation frequency in the liquid conjugation procedure. Table 2: the cytotoxic effect of *M*. *lowiana* hexane extract and methanol fraction on adult human dermal fibroblast. Figures 1-4: NMR, HRESIMS, and IR spectra for the *M. lowiana* hexane extract. Figures 5-8: NMR, HRESIMS, and IR spectra for the *M. lowiana* methanol fraction..
