# Supplementary material for: Myristica lowiana Phytochemicals as Inhibitor of Plasmid Conjugation in Escherichia coli
Source: Evid Based Complement Alternat Med. 2020 Jun 19;2020:1604638. doi: 10.1155/2020/1604638 (PMC7321511; doi:10.1155/2020/1604638)
Supplement: Supplementary Materials — Table 1: the conjugation pair, their average population size used, and their average conjugation frequency in the liquid conjugation procedure. Table 2: the cytotoxic effect of M. lowiana hexane extract and methanol fraction on adult human dermal fibroblast. Figures 1-4: NMR, HRESIMS, and IR spectra for the M. lowiana hexane extract. Figures 5-8: NMR, HRESIMS, and IR spectra for the M. lowiana methanol fraction.. [file 1604638.f1.pdf]

Supplementary Material

Myristica lowiana phytochemicals as inhibitor of plasmid conjugation in *Escherichia coli*

Awo Afi Kwapong, Sarah Soares, Stephen Ping Teo, Paul Stapleton, Simon Gibbons

Additional Experimental Detail

1. Biological activities

Table 1: The conjugation pair, their average population size used and their average conjugation frequency in the liquid conjugation procedure

**Conjugation pair**		**Average conjugation frequency ± SD (cfu/mL)**
**Average donor size ± SD (cfu/mL)**	**Average recipient size ± SD (cfu/mL)**	
pKM101 (2.19 x 10^{9} ± 7.79 x 10^{8})	ER1793 (3.77 x 10^{10} ± 3.52 x 10^{10})	2.10 x 10^{-3} ± 2.17 x 10^{-3}
TP114 (2.77 x 10^{10} ± 2.62 x 10^{10})	ER1793 (7.73 x 10^{9} ± 4.01 x 10^{9})	6.68 x 10^{-3} ± 2.78 x 10^{-3}
pUB307 (3.16 x 10^{9} ± 2.75 x 10^{9})	ER1793 (4.13 x 10^{9} ± 2.98 x10^{9})	1.45 x 10^{-1} ± 7.01 x 10^{-2}
R7K (9.17 x 10^{10} ± 5.51 x 10^{10})	JM109 (8.70 x 10^{10} ± 7.46 x 10^{10})	1.08 x 10^{-4} ± 8.65 x 10^{-5}

Table 2: The cytotoxic effect of *M. lowiana* hexane extract and methanol fraction on adult human dermal fibroblast cells (HDFa, C-013-5C)

Concentration (mg/L)	Average cells viability compared to control ± SD (%)	
	Hexane extract	Methanol fraction
12.5	111.94 ± 6.87	84.72 ± 2.80
25.0	104.99 ± 3.83	90.31 ± 3.43
50.0	107.62 ± 5.94	99.35 ± 3.59
100.0	104.91 ± 6.83	51.56 ± 5.73
200.0	101.62 ± 7.74	26.56 ± 2.21
400.0	88.52 ± 3.51	23.28 ± 1.71

2. Spectroscopic data for the *M. lowiana* hexane extract and methanol fraction

a. NMR, HRESIMS and IR spectra for the *M. lowiana* hexane extract

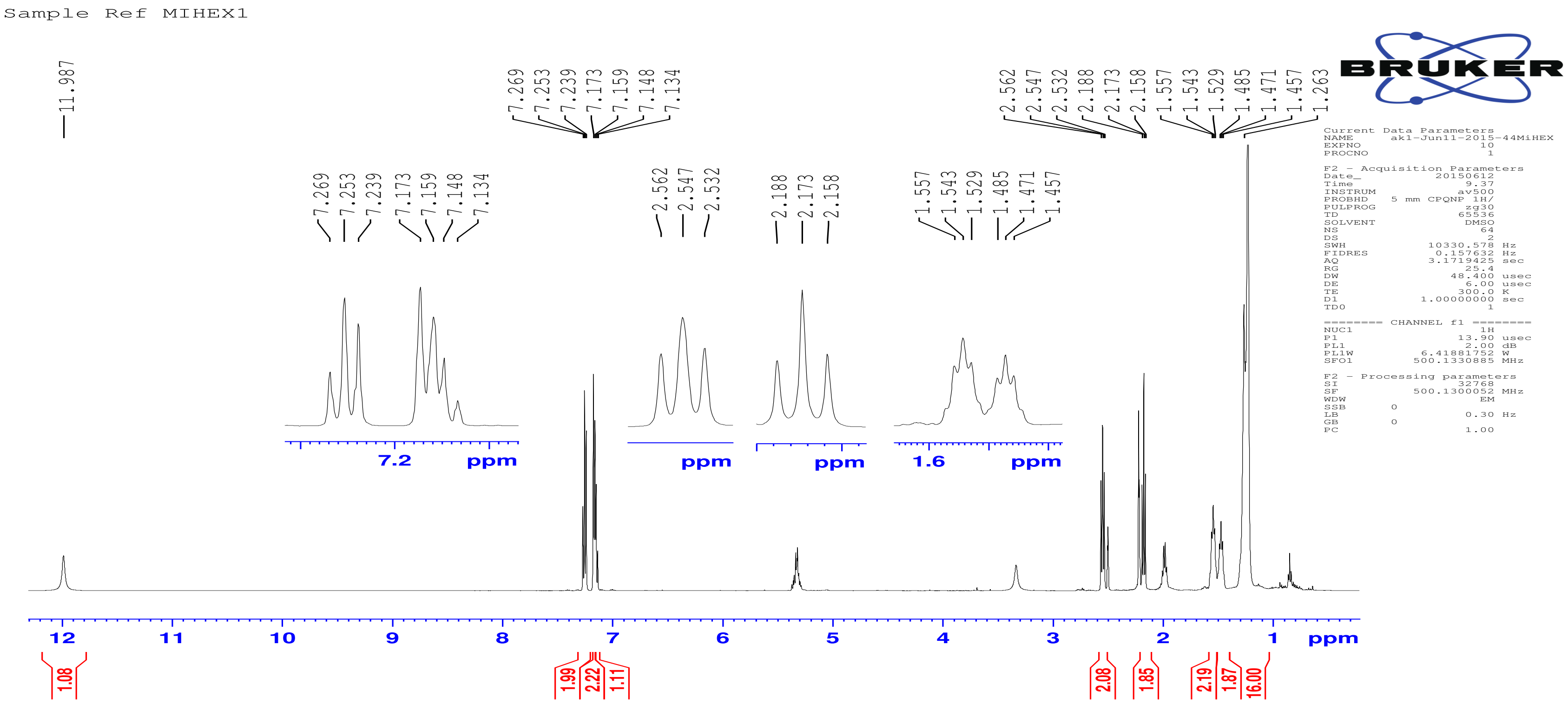

Figure 1: ^{1}**H NMR spectrum for the** ***M. lowiana*** **hexane extract, recorded in DMSO, 500 MHz**

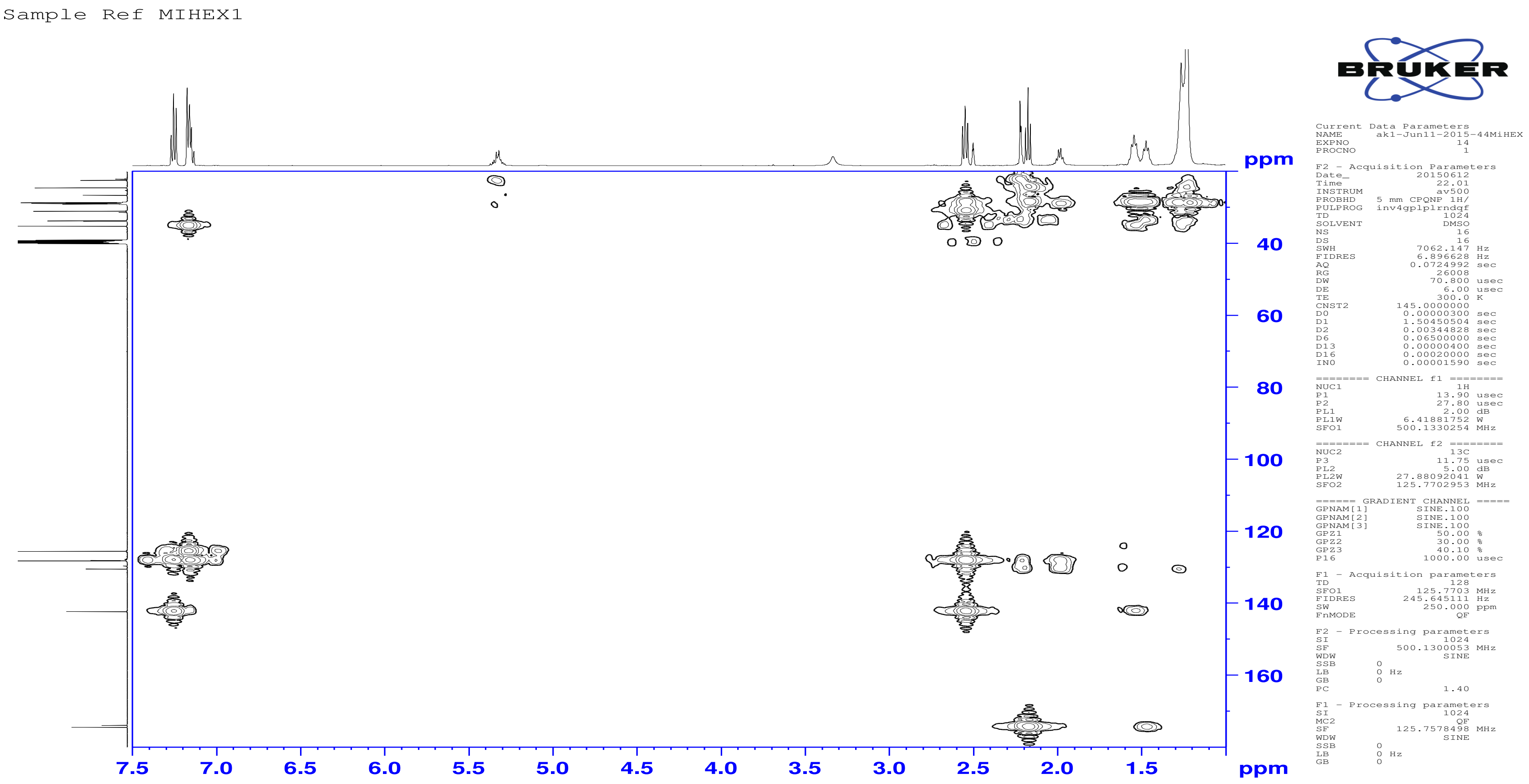

Figure 2: HMBC NMR for the *M. lowiana* hexane extract

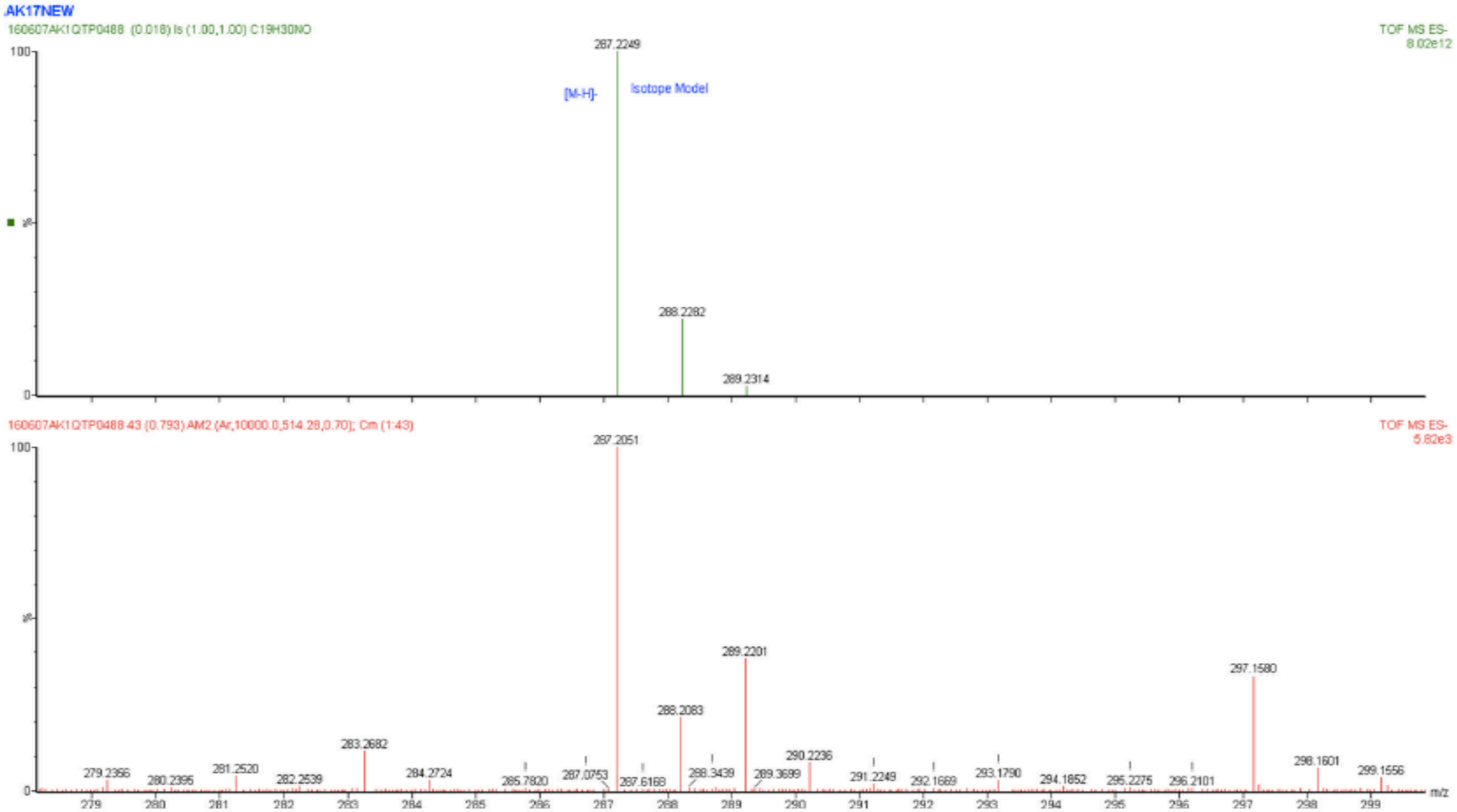

Figure 3: HRESIMS spectrum for the *M. lowiana* hexane extract

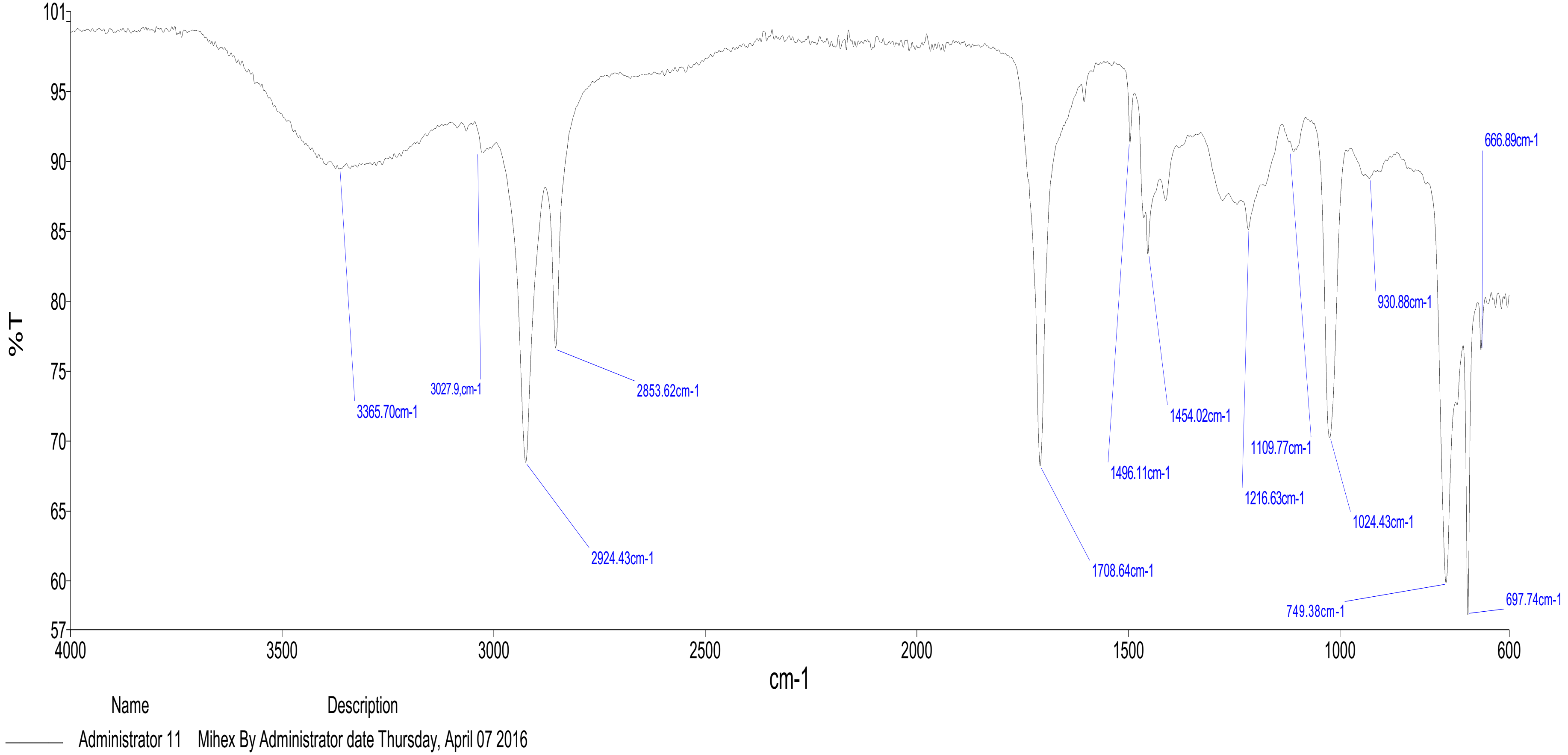

Figure 4: IR spectrum for the *M. lowiana* hexane extract

b. NMR, HRESIMS and IR spectra for the *M. lowiana* methanol fraction

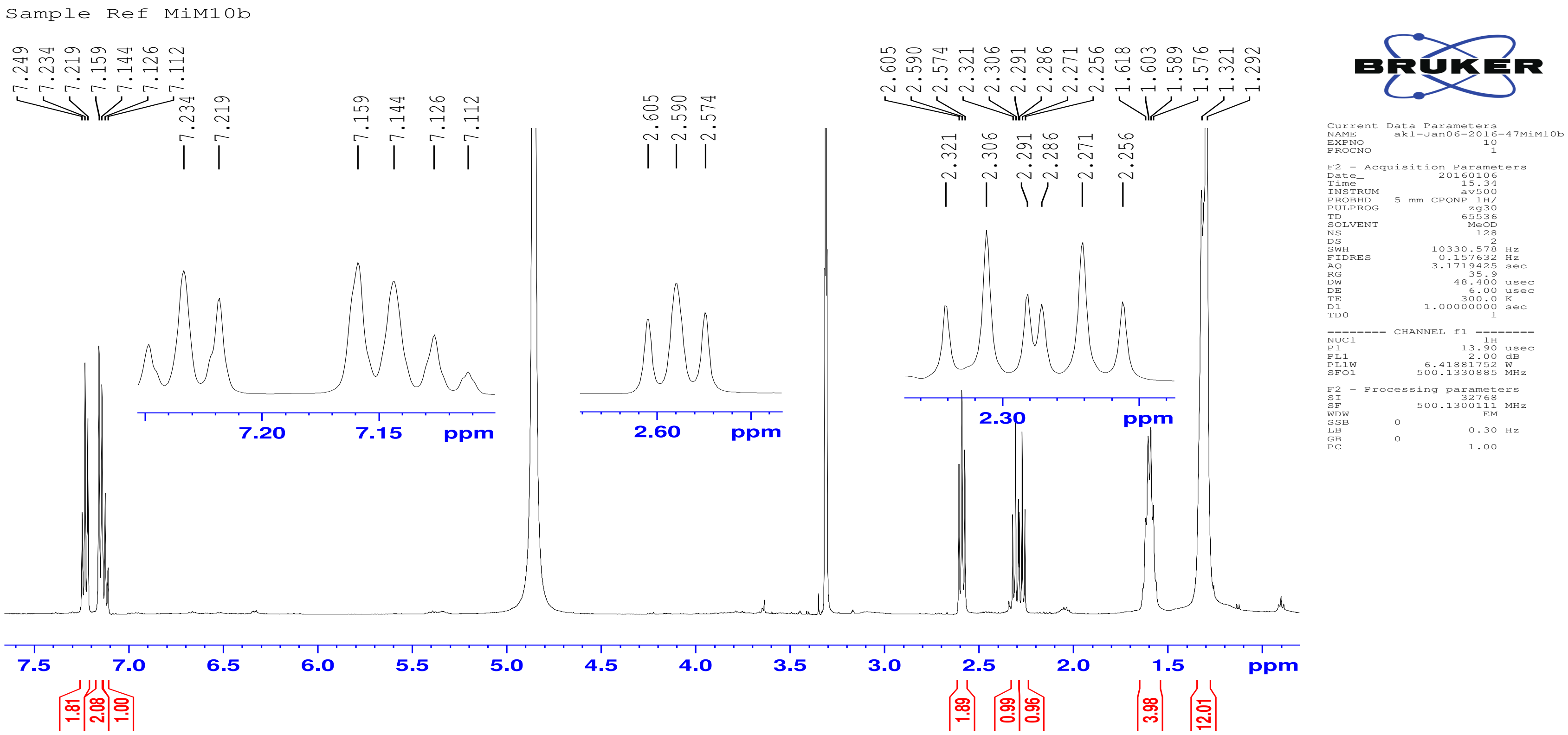

Figure 5: ^{1}H NMR spectrum for the *M. lowiana* methanol fraction, recorded in methanol, 500 MHz

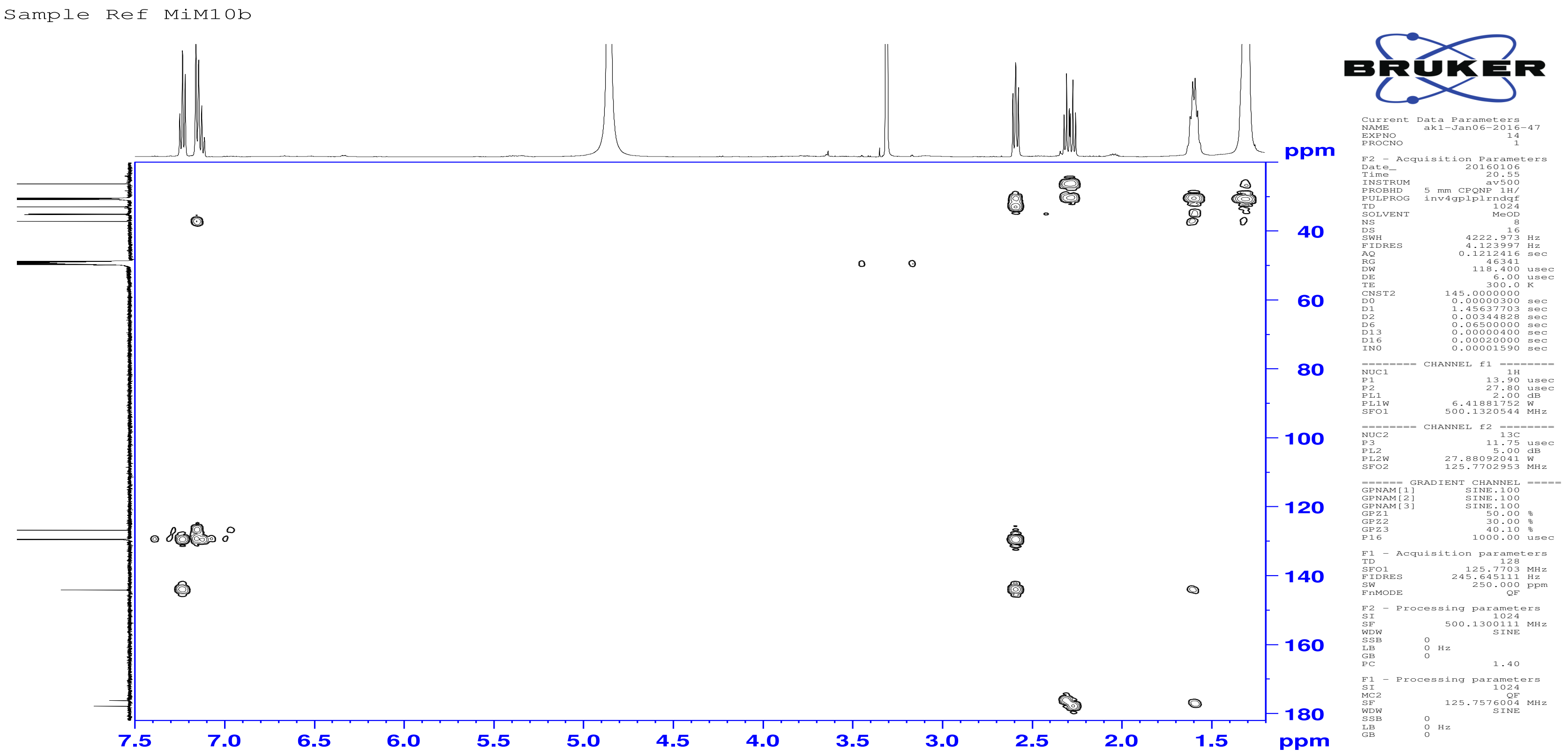

Figure 6: HMBC NMR spectrum for the *M. lowiana* methanol fraction

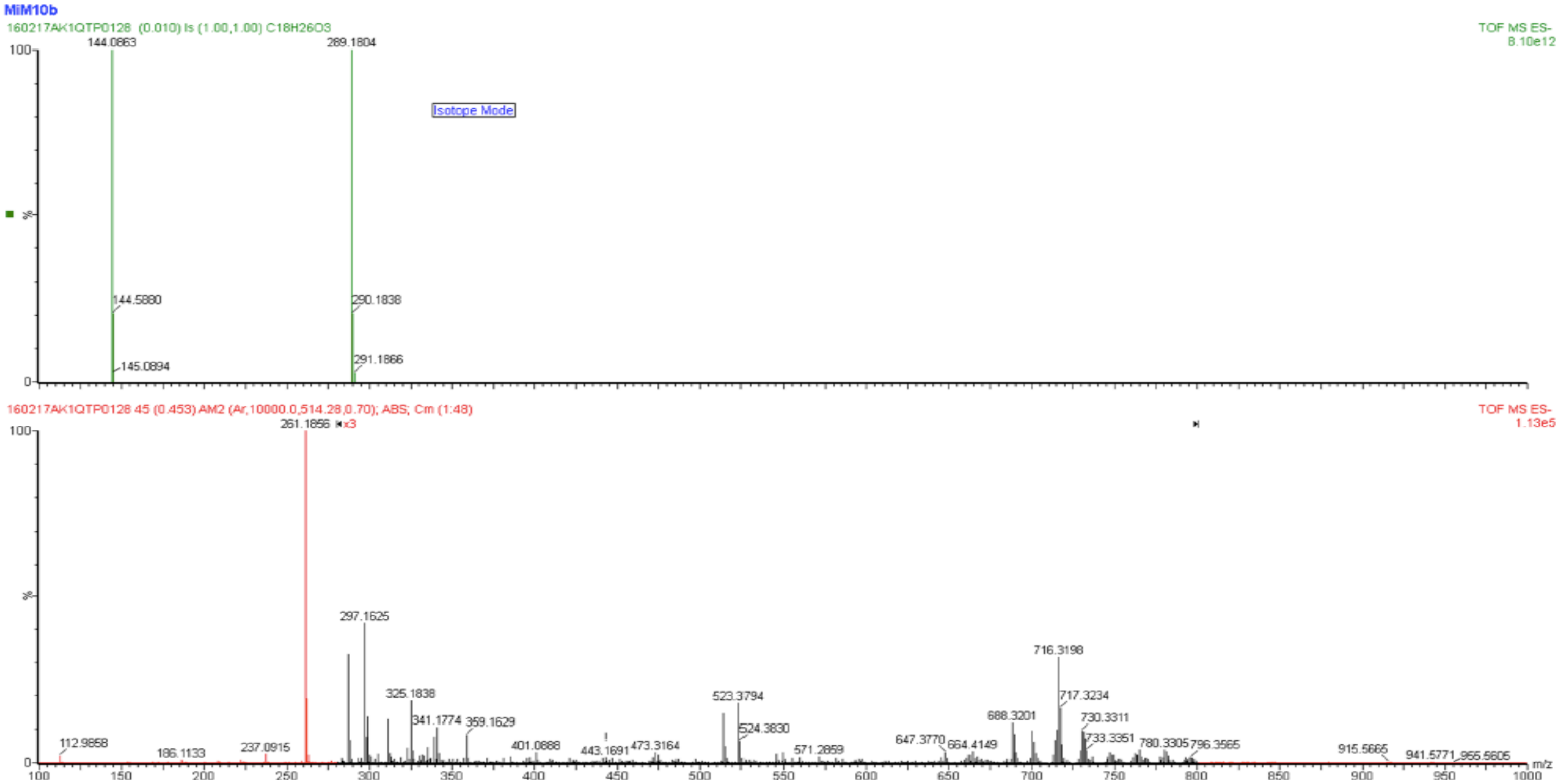

Figure 7: HRESIMS spectrum for the *M. lowiana* methanol fraction

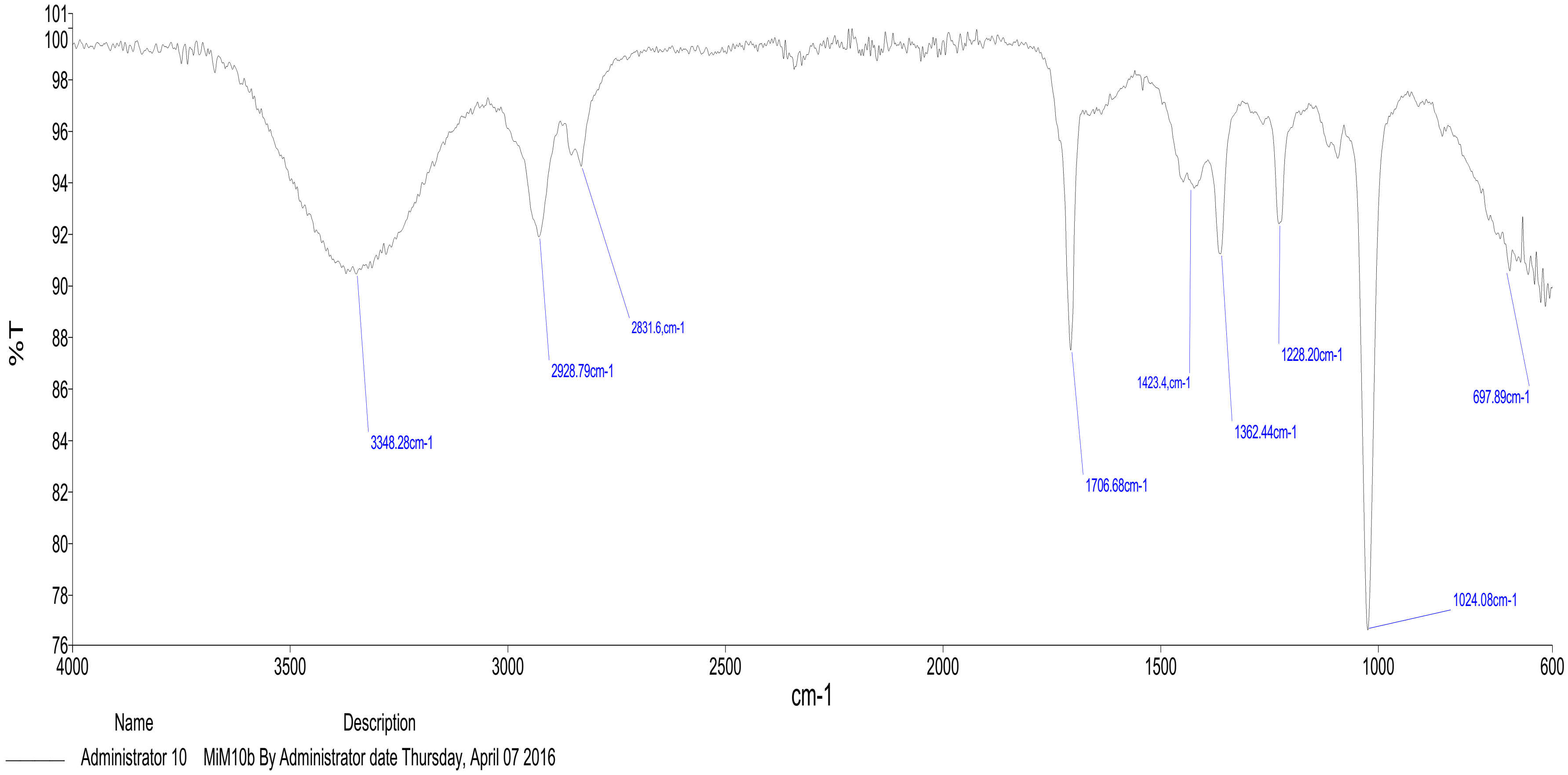

Figure 8: IR spectrum for the *M. lowiana* methanol fraction
